# On the Radical‐Induced Degradation of Quaternary Ammonium Cations for Anion‐Exchange Membrane Fuel Cells and Electrolyzers

**DOI:** 10.1002/cssc.202201571

**Published:** 2022-10-20

**Authors:** Tamas Nemeth, Thomas Nauser, Lorenz Gubler

**Affiliations:** ^1^ Electrochemistry Laboratory Paul Scherrer Institut 5232 Villigen PSI Switzerland; ^2^ Laboratory of Inorganic Chemistry ETH Zurich Vladimir-Prelog-Weg 1 8093 Zurich Switzerland

**Keywords:** anion exchange membranes, fuel cells, quaternary ammonium, radical reactions, radiolysis

## Abstract

Four benzylic‐type quaternary ammonium (QA) compounds with different electron density at the phenyl group were evaluated for their susceptibility against degradation by radicals. Time‐resolved absorption spectroscopy indicated that radicals with oxidizing and reducing character were formed upon oxidation by HO⋅ and O⋅^−^ (conjugate base of HO⋅). It was estimated that, dependent on the QA, 18–41 % of the formed radicals were oxidizing with standard electrode potentials (*E* ^0^) above 0.276 V and 13–23 % exceeded 0.68 V, while 13–48 % were reducing with *E* ^0^<−0.448 V. The stability of these model compounds against oxidation and reductive dealkylation was evaluated at both neutral and strongly alkaline conditions, pH 14. Under both conditions, electron‐donating groups promoted radical degradation, while electron‐withdrawing ones increased stability. Therefore, durability against radical‐induced degradation shows an opposite trend to alkaline stability and needs to be considered during the rational design of novel anion‐exchange membranes for fuel cells and electrolyzers.

## Graphical abstract

**Balancing act**: In anion‐exchange membranes (AEMs), a trade‐off exists between stability in alkaline media and against radical attack. Electron‐poor constituents facilitate alkaline degradation and concomitantly improve the stability against oxidizing radicals, while electron‐rich moieties have an opposite effect. The use of antioxidants in AEMs can favorably alter this sensitive balance.

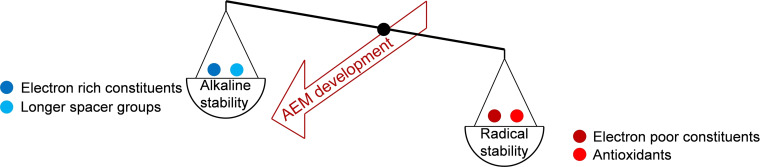

## Introduction

Transportation is responsible for a third of greenhouse gas emissions worldwide and is therefore one of the major contributing factors to global warming.^[1]^ The use of hydrogen fuel cells (FC) in this sector can help to combat the detrimental effects of climate change and anthropogenic air pollution. Anion‐exchange membranes (AEM) are of interest to substitute the traditionally used proton‐exchange membranes (PEM) in polymer electrolyte FCs owing to their (1) kinetically more facile oxygen reduction reaction (ORR) under alkaline conditions that lowers activation losses, (2) the possibility to use non‐noble metal catalysts, and (3) decreased cell component costs due to less corrosive operating conditions.^[2]^ Also, AEM water electrolyzers (AEMWE) have received substantial attention as applicable devices for sustainable hydrogen production.[^3^, ^4^] While hydrogen is commonly produced by steam reforming of hydrocarbons that results in substantial carbon dioxide emissions, water electrolyzers operate by the potentially zero‐emission process of electrochemical water splitting.^[5]^

However, the wide‐spread application of both AEMFCs and AEMWEs is severely limited due to the rapid aging of the AEM under alkaline conditions. Recent studies report that degradation is further accelerated at low hydration levels.^[6]^

Although chemical degradation of PEMs is dominated by electrophilic attack of oxidizing radicals formed during FC operation,^[7]^ in AEMFCs it is predominantly caused by the attack of OH^−^, both a strong base and a potent nucleophile, on the AEM.^[8]^

Several accepted degradation pathways exist in alkaline medium, notably: (1) Hofmann elimination, (2) nucleophilic substitution, and (3) formation of ylides that may undergo further rearrangement reactions through Stevens or Sommelet–Hauser mechanisms.^[8]^ These deleterious pathways can occur in parallel and have prompted researchers to focus on the alkaline stability improvement of AEMs. Careful design enabled to reach improved durability and high performance.^[6]^ Once membrane constituents show very high stability against base‐induced degradation (i. e., the effect of OH^−^ attack becomes negligible), ionomer stability will be determined by other detrimental processes.

Several studies describe enhanced ionomer degradation under oxygen saturation, which implies that reactive oxidizing species (ROS) influence these deleterious reactions in AEMs as well. Upon evaluating the ex‐situ stability of a trimethylammonium headgroup containing poly(*p*‐phenylene oxide)‐based AEMs, Parrondo et al. observed a significantly higher loss in ion‐exchange capacity (IEC) for O_2_‐saturated KOH solutions than for N_2_‐degassed ones.^[9]^ Nuclear magnetic resonance (NMR) experiments performed on aerated KOH solutions of the AEMs and the spin trap 5‐diisopropoxy‐phosphoryl‐5‐methyl‐1‐pyrroline‐*N*‐oxide (DIPPMPO) suggested time‐dependent formation of HO⋅ and HOO⋅ and enabled the authors to establish a possible mechanism for ROS formation. The authors’ follow‐up work involved the use of an O_2_⋅^−^‐sensitive fluorescent dye for in‐situ FC experiments.^[10]^ The rate of O_2_⋅^−^ formation showed a good correlation with the observed IEC loss.

The ex‐situ stability test performed by Espiritu et al. also shows enhanced IEC loss for vinylbenzyl chloride‐grafted low‐density polyethylene‐type AEMs in the presence of oxygen.^[11]^

Wierzbicki et al. operated a micro‐AEMFC in an electron paramagnetic resonance device that enabled to spin‐trap both HO⋅ and HOO⋅ on the cathode side and H⋅ on the anode side, in support of the original mechanism.^[12]^ However, caution needs to be exercised upon interpreting the detection of spin trap/⋅OH adduct as evidence for HO⋅ formation,^[13]^ as in exceptional cases radical adducts could be observed in the absence of HO⋅.[^14^, ^15^] Nevertheless, all these results suggest the direct involvement of radicals in AEM degradation.

**Scheme 1:**
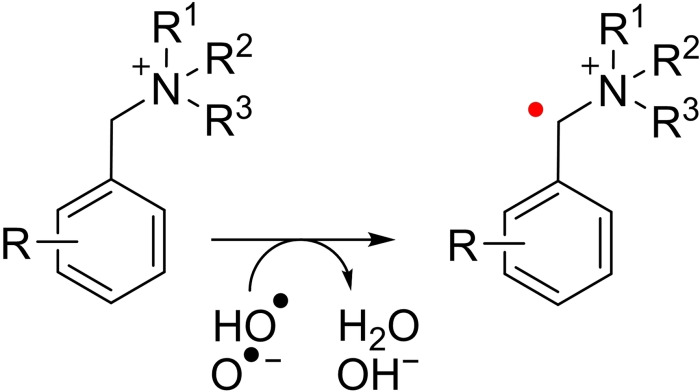
Degradation of QAs through hydrogen abstraction.

While the reaction mechanisms of OH^−^ with AEMs are well‐established,^[8]^ the effects of both HO⋅ and HOO⋅ are largely unexplored in this field. The nature and reactivity of these radicals is strongly influenced by pH; the p*K*_a_ of HOO⋅ is 4.8,^[16]^ and therefore the prevalent species under alkaline conditions is O_2_⋅^−^. A p*K*_a_ of 11.8 for HO⋅ means that both the protonated and the deprotonated form, O⋅^−^, may be present at the very alkaline pH of an operating FC.^[17]^ The radicals HOO⋅/O_2_⋅^−^ and HO⋅/O⋅^−^ are expected to abstract hydrogen from vulnerable positions (Scheme 1), although for the former these reactions are generally several orders of magnitude slower.^[16]^

**Scheme 2:**
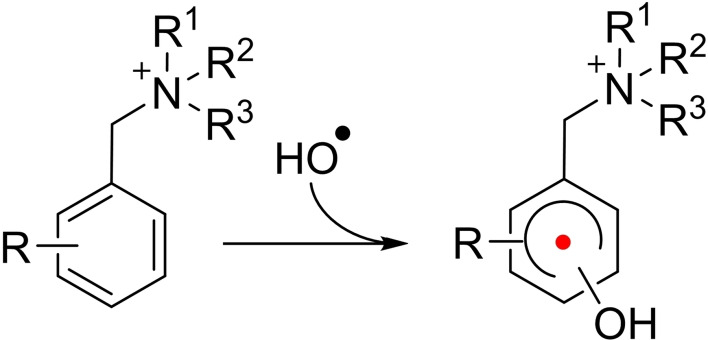
Addition of HO⋅ to QAs.

Near diffusion‐limited rates are reported for the addition of HO⋅ to aromatic compounds (Scheme 2), forming hydroxycyclohexadienyl radicals (HO‐adducts), reactive intermediates that readily undergo further reactions.^[18]^

The radical HO⋅ may also be involved in one‐electron transfer reactions, *E* ^0^(OH⋅/HO^−^)=1.9 V.^[19]^ All of these reactions are competing with each other and contribute to the degradation of QAs.

**Figure 1:**
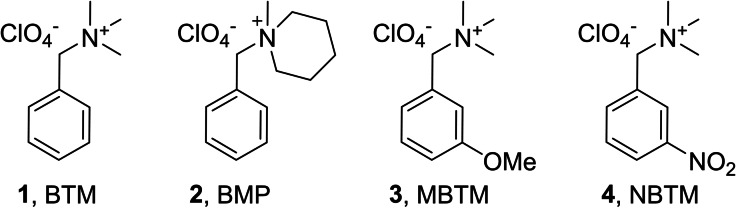
Quaternary ammonium model compounds.

It has been proposed that both HO⋅ and HOO⋅ are involved in the degradation of QAs.^[9]^ Therefore, it became our aim to better understand the complex mechanism of the radical‐induced degradation reactions of selected aromatic QA compounds, representatives of AEMs. Model compounds with benzylic groups were selected (Figure 1) that are typical building blocks of AEMs and allow direct comparison with the seminal work of Marino and Kreuer, which assessed the alkaline stability of a large number of different quaternary ammonium compounds.^[20]^ The model compounds benzyl‐trimethyl‐ammonium (BTM), *N*‐benzyl‐*N*‐methyl‐piperidinium (BMP), 3‐methoxy‐benzyl trimethyl ammonium (MBTM), and 3‐nitro‐benzyl trimethyl ammonium (NBTM), all with perchlorate as counter‐ion, account for a broad variety of electron density that is expected to heavily influence both reaction rates and lifetimes of intermediates. The use of a pulse radiolysis setup coupled with time‐resolved transient spectroscopy allowed us to study kinetics of certain radical reactions. The experimental results are further substantiated with degradation tests where radicals are produced continuously at a low concentration via gamma‐radiolysis of water.

As will be shown below, the QA structure has a large impact on the kinetics of radical‐induced degradation, and a careful balance needs to be found to develop novel AEMs that are resistant against both nucleophilic and radical attack.

## Results and Discussion

### Pulse radiolysis

We reacted the aqueous solutions of the AEM model compounds with primary radicals, produced by pulse radiolysis of water, and followed the reactions by time‐resolved UV/Vis spectrometry. Briefly, free radical chemistry is triggered when a dilute aqueous solution containing the compound of interest is exposed to nanosecond pulses of ionizing irradiation. Radiolysis of water by ionizing radiation results in the formation of primary radical species, HO⋅ (45 %), H⋅ (10 %), and e_aq_^−^ (45 %), with established yields: *G*(HO⋅)=*G*(e_aq_^−^)=0.28 μmol J^−1^ and *G*(H⋅)=0.06 μmol J^−1^ [Eq. (1)] at neutral pH.^[18]^ These yields are also correct for dilute aqueous solutions. In case of neutral solutions, under N_2_O‐saturation (24.8 mm at 20 °C) solvated electrons can be converted into HO⋅ [Eq. (2), *k*=9.1×10^9^ m ^−1^ s^−1^], thus increasing the yield to *G*(HO⋅)=0.56 μmol J^−1^.[^21^, ^22^] Under these conditions, the contribution of H⋅ to the reactions is ≤10 % and is, therefore, oftentimes neglected.
(1)

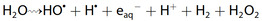

(2) $$N{}_{2}O+H{}_{2}O+e{}_{\mathrm{aq}}{}^{-}\to N{}_{2}+\mathrm{HO}{}^{•}+\mathrm{OH}{}^{-}$$

**Figure 2:**
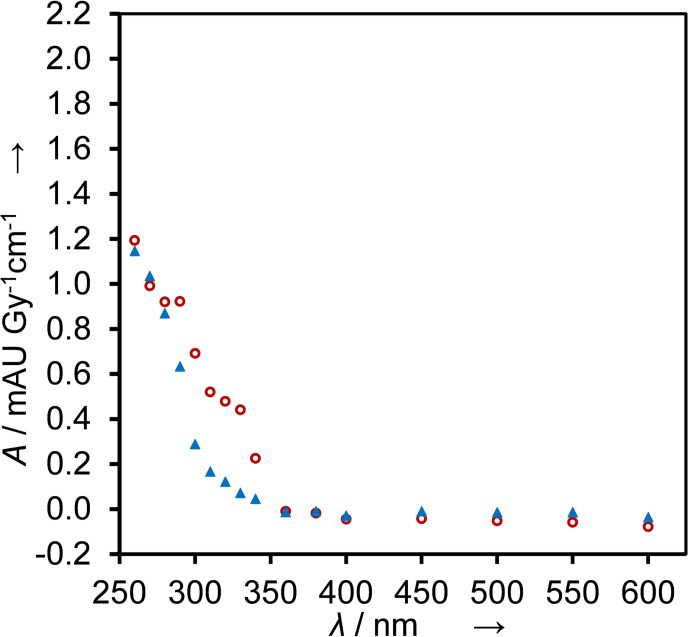
Transient absorption spectrum 10 (red circles) and 100 μs (blue triangles) after the pulse (dose 5–20 Gy), obtained from time‐resolved absorbance readings, normalized to 1 Gy and to the optical path length, measured in N_2_O‐saturated solutions that contained 0.1 mm BTM and 10 mm KPi buffer.

When we irradiated N_2_O‐saturated solutions of 100 μm BTM in 10 mm KPi buffer (pH 7) reactions initiated by HO⋅ dominated (≈90 %). With doses of 5–20 Gy radical concentrations between 3 and 12 μm are produced. Kinetics traces were recorded at wavelengths between 260 and 600 nm (Figure 2; see Figure S1 for traces of BMP, MBTP, and NBTM).

The transient absorption spectrum recorded 100 μs after the pulse indicates that the formed intermediates are persistent (*t*_1/2_>100 μs) and absorb in the UV part of the spectrum.

Under strongly alkaline conditions (0.1 m KOH, pH 13), additional reactions are taken into account [Eqs. (3) and (4), with *k*=1.2×10^10^ and 2.2×10^7^ m ^−1^ s^−1^, respectively]:

(3) $$\mathrm{HO}{}^{•}+\mathrm{OH}{}^{-}\to O{}^{•}{}^{-}+H{}_{2}O$$

(4) $$H{}^{•}+\mathrm{OH}{}^{-}\to e{}_{\mathrm{aq}}{}^{-}+H{}_{2}O$$

**Figure 3:**
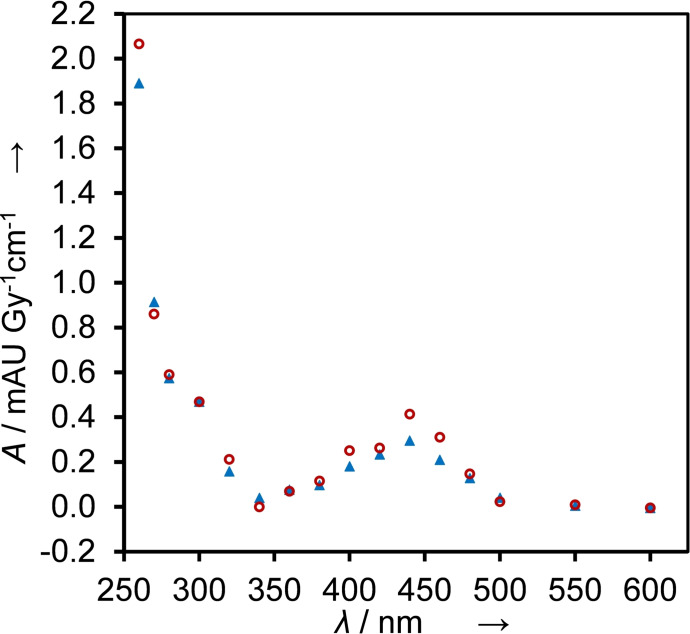
Transient absorption spectrum 10 (red circles) and 100 μs (blue triangles) after the pulse (dose 5–20 Gy), obtained from time‐resolved absorbance readings, normalized to 1 Gy and to the optical path length, measured in N_2_O‐saturated solutions that contained 0.1 mm BTM and 100 mm KOH.

Both HO⋅ (p*K*_a_=11.8) and H⋅ react with OH^−^ to form O⋅^−^ and e_aq_^−^, respectively.^[18]^ Therefore, in N_2_O‐saturated solutions the yield of O⋅^−^ corresponds to *G*(O⋅^−^)=0.55 μmol J^−1^, while *G*(HO⋅) decreases to 0.06 μmol J^−1^ (see also the Supporting Information for more details on radical yields). Hydrogen abstraction dominates the reactivity of O⋅^−^ (Scheme 1), electron transfer or addition is of minor relevance here. Reactions of HO⋅ contribute around 10 % to the observed absorbance changes. The spectrum shown in Figure 3 is therefore caused by multiple species formed by H⋅‐abstraction and HO⋅ addition. In contrast to measurements at pH 7 (Figure 2), at pH 13 a persistent new species is detected with an absorption maximum at 450 nm and a lifetime in the millisecond range (Figures 3 and S2).

The electron‐withdrawing trimethylammonium headgroup in BTM may lower the p*K*_a_ of the allyl alcohol analogous HO‐adduct intermediates to values where deprotonation of the HO‐group also needs to be considered [Eq. (5)]. This also contributes to the complex component spectrum.

(5) $$\left(\mathrm{ArCH}{}_{2}\mathrm{NR}{}_{3}{}^{+}\cdot \cdot \cdot \mathrm{OH}\right){}^{•}\to \left(\mathrm{ArCH}{}_{2}\mathrm{NR}{}_{3}{}^{+}\cdot \cdot \cdot O\right){}^{•}{}^{-}+H{}^{+}$$

We observed similar absorbance changes for all the other model compounds (Figure S1), the piperidinium‐derivative BMP, the methoxy‐ and nitro‐substituted MBTM and NBTM, respectively.

**Figure 4:**
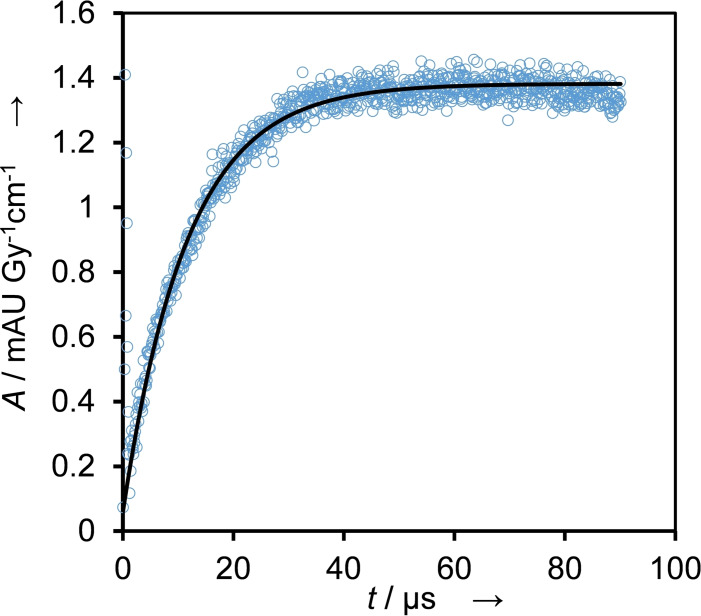
Reaction with the indicator for oxidants, ABTS^2−^, performed in N_2_O‐saturated solution containing 1 mm BTM, 0.1 mm ABTS^2−^, and 100 mm KOH. Evaluated at 650 nm (blue circles) by performing a first‐order fit on the dataset (black line), *k*_obs_=8.6×10^4^ s^−1^.

With the intention of establishing mass balance, we attempted to obtain quantitative information on the formed free radicals by performing “redox titration”. The rapid build‐up of absorbance indicates that reactions involving both O⋅^−^ and HO⋅ were completed in around 3 μs (see Figure S3), therefore the pseudo first order rate constant for the build‐up is *k*_obs, build‐up_≈5×10^5^ s^−1^. Consequently, every reaction with a slower build‐up is rate determining and enables to conveniently monitor these reactions. First, we used 2,2’‐azino‐bis(3‐ethylbenzothiazoline‐6‐sulfonic acid) diammonium (ABTS^2−^) to quantify oxidizing radicals with standard electrode potentials exceeding 0.68 V [*E* ^0^(ABTS⋅^−^/ABTS^2−^)=0.68 V].[^23^, ^24^] Electron transfer from ABTS^2−^ to oxidizing radicals resulted in a rapid absorbance build‐up at 650 nm (Figure 4), as shown in Equation (6), where only the formed ABTS⋅^−^ absorbs. We obtained a pseudo‐first‐order rate constant of *k*_6,obs,BTM_=8.6×10^4^ s^−1^ from performing a pseudo‐first‐order fit on the dataset, dividing it with the concertation ABTS^2−^ (0.1 mm) yields *k*_6,BTM_≈8.6×10^8^ m ^−1^ s^−1^, the rate constant for Equation 6.

(6) $$\mathrm{ABTS}{}^{2-}+\mathrm{oxidizing}\;\mathrm{radicals}\to \mathrm{ABTS}{}^{•}{}^{-}+\mathrm{product}$$

The yield of oxidizing radicals can be calculated by first quantifying the concentration of formed ABTS⋅^−^ from the observed absorbance maximum at 650 nm using its known molar absorbance coefficient and then comparing it with the theoretically possible concentration of formed radicals (see details in the Supporting Information). For BMT this equals to around 22 %.

Oxidizing radicals were further probed using *N,N,N‘,N‘*‐tetramethyl phenylenediamine (TMPD), which has a redox potential of *E* ^0^(TMPD⋅^+^/TMPD)=0.276 V, and therefore is able to detect oxidizing species with relatively low oxidation potential [Eq. 7].^[25]^

(7) $$\mathrm{TMPD}+\mathrm{oxidizing}\;\mathrm{radicals}\to \mathrm{TMPD}{}^{•}{}^{+}+\mathrm{product}$$

**Figure 5:**
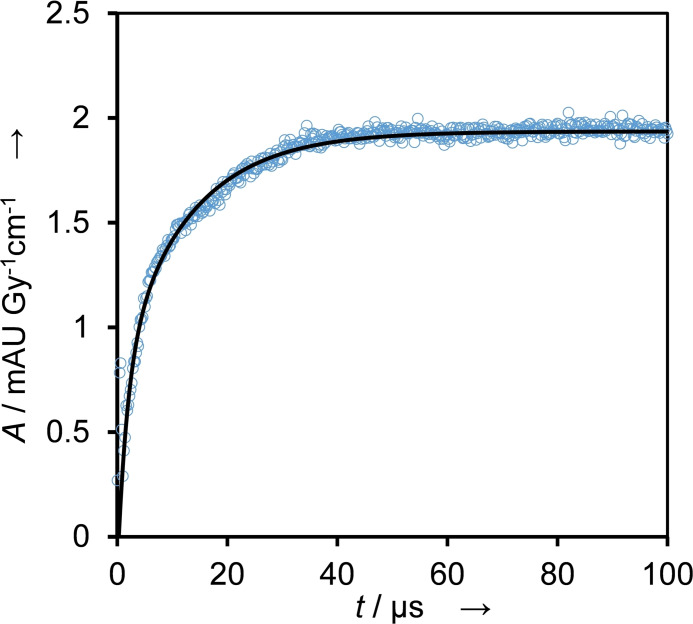
Reaction with the indicator for oxidants, TMPD, performed in N_2_O‐saturated solution containing 1 mm BTM, 0.1 mm TMPD, and 100 mm KOH. Evaluated at 612 nm (blue circles) by performing a double exponential fit on the dataset (black line), *k*_7,obs,BTM,A_=4.4×10^5^ s^−1^ and *k*_7,obs,BTM,B_=6.4×10^4^ s^−1^.

The dataset (Figure 5) could be satisfactorily fitted with a double exponential function, indicating that more than one oxidizing species are present. Pseudo‐first‐order rate constants of *k*_7,obs,BTM,A_=4.4×10^5^ s^−1^ and *k*_7,obs,BTM,B_=6.4×10^4^ s^−1^ were obtained, with component A dominating as indicated by the ratio of *k*_7,obs,A_/*k*_7,obs,B_≈4.5. The rate constants for Equation (7) are therefore *k*_7,BTM,A_≈4.4×10^9^ m ^−1^ s^−1^ and *k*_7,BTM,B_≈6.4×10^8^ m ^−1^ s^−1^, respectively. We estimated the yield of radicals capable of oxidizing TMPD as around 27 %.

The fact that we could not establish total mass‐balance by neither of the two experiments indicates that species with reducing properties are present that consume the formed oxidized products ABTS⋅^−^ and TMPD⋅^+^.

We attempted to give an estimate on their yield by performing redox titration using the indicator methyl viologen (MV^2+^), which has a redox potential of *E* ^0^(MV^2+^/MV⋅^+^)=‐0.448 V.^[26]^ Electron transfer to MV^2+^ takes place according to Equation 8:

(8) $$\mathrm{MV}{}^{2+}+\mathrm{reducing}\;\mathrm{radicals}\to \mathrm{MV}{}^{•}{}^{+}+\mathrm{product}$$

**Figure 6:**
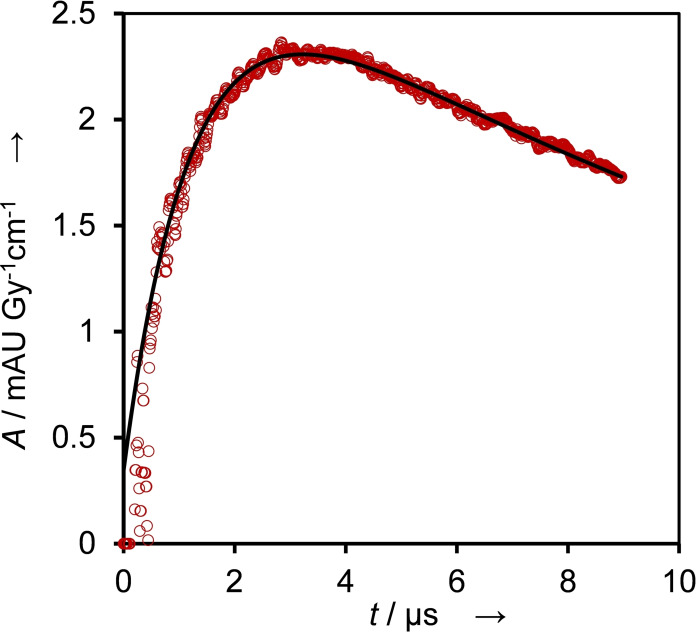
Reaction with the indicator for reductants, MV^2+^, performed in N_2_O‐saturated solution containing 1 mm BTM, 0.1 mm MV^2+^, and 100 mm KOH. Evaluated at 600 nm (red circles) by performing a fit on the dataset assuming consecutive first‐order reactions (black line), *k*_8,obs,BTM,build‐up_=8.0×10^5^ s^−1^ and *k*_8,obs,BTM,decay_=8.1×10^4^ s^−1^.

Decay of the formed MV⋅^+^ can be observed already at 3 μs after the pulse (Figure 6). It suggests that the primary reactions of BTM with O⋅^−^ and HO⋅ are not separated well enough in time from the secondary reactions involving MV^2+^ [Eq. (8)]. Therefore, the calculated yield of reducing radicals is a conservative lower estimate. We performed fitting with a model of consecutive first‐order reactions where the intermediate species is observed. We obtained pseudo‐first‐order rate constants of *k*_8,obs,BTM,build‐up_=8.0×10^5^ s^−1^ and *k*_8,obs,BTM,decay_=8.1×10^4^ s^−1^, therefore the rate constants for Equation (8) are *k*_8,BTM,build‐up_≈8.0×10^8^ m ^−1^ s^−1^ and *k*_8,BTM,decay_≈8.1×10^7^ m ^−1^ s^−1^, respectively.

We estimated the yield of radicals stemming from BTM capable of reducing MV^2+^ as around 48 %. With the other model compounds, BTM, MBTM, and NBTM, the rapid consumption of the formed MV⋅^+^ was already prominent approximately 2 μs after the pulse (data not shown), resulting in further underestimation in the yield of reducing species. In addition, this precluded us from determining the pseudo‐first‐order rate constants of build‐up and decay [Eq. (8)] for BMP, MBTM, and NBTM. Table 1 lists the yields of formed radicals for each model compound, and the respective observed rates of formation are given in Table 2.

**Table 1:** Yield of formed oxidizing and reducing radicals under alkaline conditions (pH 13).

Compound	Yield (ox.) *E* ^0^>+0.680 V [%]	Yield (ox.) *E* ^0^>+0.276 V [%]	Yield (red.) *E* ^0^<−0.448 V [%]
BTM	22.3±0.3	26.9±2.2	48.4±2.8
BMP	23.3±1.7	41.0±4.5	20.9±3.2
MBTM	10.4±0.3	37.2±0.3	19.4±1.6
NBTM	13.1±0.2	18.4±0.5	13.3±1.5

**Table 2:** Observed pseudo‐first‐order rates of formation.

Compound	*k*_6,obs_ [s^−1^]	*k*_7,obs_ (component A or B) [s^−1^]	*k*_8,obs_ [s^−1^]
BTM	8.6×10^4^	4.4×10^5^ (A) 6.4×10^4^ (B)	8.0×10^5^
BMP	5.3×10^4^	1.4×10^4^ (A) 3.5×10^5^ (B)	–
MBTM	8.0×10^4^	6.9×10^4^ (A) 1.0×10^4^ (B)	–
NBTM	8.1×10^4^	5.2×10^4^ (A) 1.3×10^6^ (B)	–

### Stability assessment through product analysis

We showed above that, upon oxidative attack by HO⋅ and O⋅^−^, radical intermediates that are oxidizing and reducing in nature are formed and that different species are present at neutral and under alkaline conditions. Therefore, in an attempt to evaluate the radical stability of the model compounds we irradiated dilute aqueous solutions with γ‐radiation from a ^60^Co source to form radicals by water radiolysis [Eq. (1)]. In order to focus purely on radical‐induced degradation, we performed the irradiation at room temperature. Control experiments showed no degradation for the model compounds at this temperature in the timescale of our experiments, hydrolysis by OH^−^ is therefore negligible.

In contrast to pulse radiolysis, here the radicals are formed at a continuous low rate that is known to resemble better the conditions in a FC or electrolyzer.^[27]^ N_2_O‐, air‐, or argon‐saturated solutions of 1 mm model compound at pH 7 (10 mm KPi) or at pH 14 (1 m KOH) were irradiated and the dose‐dependent degree of degradation was quantified by HPLC‐UV (Tables 3 and 4 contain the compiled data). We multiplied the irradiation dose (1 Gy=1 J kg^−1^≈1 J L^−1^) by the known radiochemical yield of primary radicals (*G*=0.62 μmol J^−1^) to obtain the quantities of formed primary radicals, expressed in μm.

**Table 3:** Degree of degradation expressed in μM of N_2_O‐, air‐, or Ar‐saturated solutions of 1 mm BTM, BMP, MBTM, or NBTM as a function of formed primary radicals. pH 7 was established by 10 mm KPi buffer.

Compound	Gas	Formed primary radicals [μm]			
		124	248	496	744
BTM (pH 7)	N_2_O	74	124	204	308
	air	35	59	165	278
	Ar	126	186	321	461
BMP (pH 7)	N_2_O	37	75	147	229
	air	48	104	199	296
	Ar	133	208	347	463
MBTM (pH 7)	N_2_O	100	161	314	443
	air	74	146	282	398
	Ar	113	188	348	533
NBTM (pH 7)	N_2_O	10	18	30	51
	air	10	13	15	27
	Ar	10	24	54	84

**Table 4:** Degree of degradation expressed in μM of N_2_O‐, air‐, or Ar‐saturated solutions of 1 mm BTM, BMP, MBTM, or NBTM as a function of formed primary radicals. pH 14 was established by 1 m KOH.

Compound	Gas	Formed primary radicals [μm]			
		124	248	496	744
BTM (pH 14)	N_2_O	146	212	297	431
	air	50	128	271	375
	Ar	122	224	379	537
BMP (pH 14)	N_2_O	165	289	455	707
	air	119	209	347	419
	Ar	145	245	414	555
MBTM (pH 14)	N_2_O	173	279	495	726
	air	121	240	422	520
	Ar	152	253	423	569
NBTM (pH 14)	N_2_O	62	132	229	334
	air	60	110	169	239
	Ar	91	120	226	328

In contradiction to expectations, degradation was significantly reduced in the presence of oxygen. Degradation yields were strongly dependent on functional groups at the phenyl ring. Damage by reductive radicals (argon saturation) is in most cases equally detrimental as damage by oxidative radicals (N_2_O saturation). The degradation was substoichiometric under neutral conditions, while at pH 14 we observed a superstoichiometric damage at low irradiation doses. This is in stark contrast to measurements for PEM analogous model compounds.^[27]^ Below we give a detailed account of the observations.

### Influence of the nature of primary radical species on degradation

Systematically lower degradation was observed for air‐saturated solutions than for N_2_O‐ or argon‐saturated ones. This is primarily related to the yields and nature of radicals formed following γ‐radiolysis of water [Eq. (1)]. Due to N_2_O‐saturation, at neutral pH solvated electrons are almost fully (96 %) converted to HO⋅ via Equation (2), *G*(HO⋅)=0.55 μmol J^−1^;[^21^, ^22^] while at pH 14 a near‐quantitative deprotonation of the formed HO⋅ yields O⋅^−^.^[17]^ At this pH the primary radical H⋅ is quantitatively converted to e_aq_^−^ [Eq. (4)], further increasing the final yield of O⋅^−^ to 0.55+0.06=0.61 μmol J^−1^. Therefore, the reactions under N_2_O are dominated by either HO⋅ or by its deprotonated form, O⋅^−^. In the case of argon‐saturation, in absence of a conversion reaction for e_aq_^−^, the yield of the primary radicals HO⋅ and e_aq_^−^ equals 0.28 μmol J^−1^ at pH 7. At pH 14 we again have to consider the conversion of H⋅ to e_aq_^−^ and can therefore estimate *G*(e_aq_^−^) as 0.28+0.06=0.34 μmol J^−1^ [Eq. (4)]. When we substitute argon with oxygen the formed e_aq_^−^ are partially scavenged by the biradical O_2_ [Eq. (9)] to yield O_2_⋅^−^ with *k*=1.9×10^10^ m ^−1^ s^−1^:^[16]^

(9) $$O{}_{2}+e{}_{\mathrm{aq}}{}^{-}\to O{}_{2}{}^{•}{}^{-}$$

Our data convincingly shows that oxidation of AEMs by O_2_⋅^−^ is significantly less detrimental than by HO⋅ and O⋅^−^ (Tables 3 and 4). This is in line with expectations, as reactions of electron‐rich compounds with HO⋅ and O⋅^−^ are typically at least an order of magnitude faster than with O_2_⋅^−^.[^16^, ^18^] On the other hand, quaternary amines are potent electrophiles and therefore extremely susceptible to reduction by e_aq_^−^, resulting in fragmentation [Eqs. (10) and (11), with *k*=1.0×10^10^ m ^−1^ s^−1^ for both reactions].^[28]^ The lower degrees of degradation under air‐saturation are the direct consequences of these combined effects.

(10) $$\mathrm{ArCH}{}_{2}\mathrm{NR}{}_{3}{}^{+}+e{}_{\mathrm{aq}}{}^{-}\to \mathrm{ArCH}{}_{2}\mathrm{NR}{}_{2}+R{}^{•}$$

(11) $$\mathrm{ArCH}{}_{2}\mathrm{NR}{}_{3}{}^{+}+e{}_{\mathrm{aq}}{}^{-}\to \mathrm{ArCH}{}_{2}{}^{•}+\mathrm{NR}{}_{3}$$

The change in radical distribution under air can be calculated by multiplying the reported rate constants (*k*) by the solute concentration, and the obtained pseudo‐first‐order rate constants (*k’*) can be directly used to estimate the yields (Table 5). Therefore, in the presence of oxygen, e_aq_^−^ available to react with the model compounds decreases at pH 7 to 0.188 μmol J^−1^, while at pH 14 to 0.228 μmol J^−1^. Concomitantly, the yield of O_2_⋅^−^ changes to 0.092 μmol J^−1^ (pH 7) and to 0.112 μmol J^−1^ (pH 14).

**Table 5:** Fate of the hydrated electrons dependent on reaction conditions.

Reaction	*k* [m ^−1^ s^−1^]	Conc. [mm]	*k‘* [s^−1^]	Yield [%]
(2)	9.1×10^9^	24.8	2.2×10^8^	96^[a]^
(9)	1.9×10^10^	0.26	4.9×10^6^	33^[b]^
(10) or (11)	1.0×10^10^	1	1.0×10^7^	4^[a]^ or 67^[b]^

[a] Under N_2_O‐saturation. [b] Under air‐saturation.

### Effect of pH

Consistently higher degradation was observed at pH 14 than at pH 7. This can be rationalized by the higher yield of e_aq_^−^ at elevated pH, and, also, by the differences in reactivity between HO⋅ (pH 7) and O⋅^−^ (pH 14). While HO⋅ will predominantly form hydroxycyclohexadienyl radicals (Scheme 2), O⋅^−^ will primarily abstract hydrogens from vulnerable positions, which has a more detrimental effect as oxygen may react with these H‐abstracted radical intermediates forming peroxyl radicals [Eq. (12)]. These reactive oxidative species may serve as additional sources of radicals.

(12) $$\mathrm{ArCH}{}^{•}\mathrm{NR}{}_{3}{}^{+}+O{}_{2}\to \mathrm{ArCHOO}{}^{•}\mathrm{NR}{}_{3}{}^{+}$$

Although similar rate constants are reported for the reaction of e_aq_^−^ (argon saturation) and HO⋅ (N_2_O saturation) with benzyltrialkylammonium cations,^[29]^ we observed at pH 7 a more pronounced degradation under argon‐saturation than in N_2_O. There is nothing known about the mechanism of the pH‐dependence of follow‐up reactions. We may interpret these observations as a result of the differences in overall stabilities of the formed radical intermediates. Under alkaline conditions the opposite could be detected for BMP, MBTM, and NBTM: attack by O⋅^−^ proved to be more deleterious than that by e_aq_^−^.

### Impact of electron density

The seminal work of Marino and Kreuer addressed the alkaline stability of quaternary ammonium cations.^[20]^ The authors found that electrophilicity of the QAs directly influences alkaline stability: the presence of the electron‐withdrawing nitro group (−I effect) in NBTM enhances its electrophilic character and therefore facilitates the nucleophilic attack of OH^−^. In contrast, the methoxy group (+I and +M effect) in MBTM and the piperidino head group (+I) in BMP have an opposite effect by lowering the overall electrophilicity.

The presence of a methoxy or piperidino group could not effectively suppress degradation. In fact, MBTM had the lowest stability under all experimental conditions (Tables 3 and 4). Under neutral conditions the observations can be rationalized by the fact that, unlike OH^−^, HO⋅ is electrophilic. Consequently, every modification that decreases the electron density of a QA will reduce the rate of attack by HO⋅. This is confirmed by the around factor 5 higher stability of the nitro‐substituted NBTM. At pH 14 we expect hydrogen abstraction to dominate in N_2_O‐saturated solutions, complemented by reductive dealkylation in air‐ or argon‐saturated solutions. Again, NBTM showed the lowest degree of degradation, being nearly a factor of two more stable.

To further substantiate the results, we used density functional theory (DFT) calculations to estimate homolytic bond dissociation enthalpies (BDE) that correspond to the ease of H⋅ abstraction from the QAs. Results show that the weakest hydrogen bond is unambiguously the benzylic one and that MBTM is the most vulnerable due to its comparatively lower BDEs, while NBTM has the highest stability (Table 6). In case of MBTM, the electron‐donating methoxy group stabilizes the formed radical center, and in the piperidinium‐derivative BMP, the methylene groups impose a similar effect.^[30]^ Meanwhile, the relatively electron‐poor character of NBTM destabilizes the formed radical intermediates.

**Table 6:** Calculated bond dissociation enthalpies of QAs.

Position	BDE [kJ mol^−1^]			
	BTM	BMP	MBTM	NBTM
benzyl	404.2	402.6	402.0	408.2
methyl	449.0	439.0	447.8	448.9
methoxy	–	–	415.3	–
*N*‐methylene	–	429.7	–	–

### Implications for AEMs

Changes in electron density through structural modifications have an apparent effect on stability: the presence of an electron‐withdrawing group decreases alkaline stability by lowering the overall electrophilic character. Concomitantly, stability against radical attack is increased by the reduced stabilities of the formed intermediate radical species. Therefore, a careful balance needs to be established between alkaline and radical durability. Sterically protected polyarylimidazoliums and electron‐rich polynorbornenes as backbones for AEMs are in the center of intense attention, as they possess high IEC and exceptional stability under alkaline conditions.[^6^, ^31^, ^32^] However, electron‐rich ionomers based on these designs are more susceptible to hydrogen atom abstraction by O⋅^−^ or to dealkylation through reduction.^[33]^ In case of PEMs, the generation of H⋅ by hydrogen splitting on the catalyst is reported.^[34]^ This preferentially takes place on the anode side but may also manifest on the cathode side, as a result of fuel crossover. Analogously, this can occur in AEMs if a noble metal catalyst is used.^[12]^ Under alkaline conditions the formed H⋅ will deprotonate and yield highly reducing hydrated electrons [Eq. (4)]. We could establish through pulse radiolysis experiments that reducing species are also formed following the attack of O⋅^−^ (Figure 6 and Table 1) that may infer a similarly detrimental effect as e_aq_^−^. It has been reported that OH^−^‐induced degradation of AEM constituents becomes more pronounced at reduced levels of hydration.^[6]^ Similarly, a change in relative humidity will alter the stability and reactivity of radicals and the formed intermediates. Its consequences are presently unknown and need to be evaluated in detail. All of these effects need to be considered during the rational design of novel AEMs.

### Comparison of stability between AEMs and PEMs

We recently reported on the stability of monomer analogous compounds of aromatic hydrocarbon‐based PEMs against the attack of HO⋅.^[27]^ We concluded that electron density has a decisive impact on stability of PEMs as we observed superstoichiometric degradation in case of an electron‐poor model compound. The radical HO⋅ reacts essentially diffusion‐controlled with aromatic PEM constituents, precluding its effective scavenging by an antioxidant.[^35^, ^36^] PEMs are generally unreactive towards reducing species due to their electron‐poor character, resulting from the strongly electron‐withdrawing proton‐conductive sulfonate groups. In comparison, we demonstrated here that positively charged AEMs are highly vulnerable to both reduction and hydrogen abstraction. On the other hand, we observed superstoichiometric degradation of the AEM model compounds only in a few instances (Tables 3 and 4), suggesting their higher overall stability against radical attack compared to PEMs. A lower reactivity towards HO⋅ and O⋅^−^ enables the use of radical scavengers for damage mitigation in AEMs, as shown in a recent publication.^[37]^ Therefore, even if currently high alkaline stability is prioritized, the concept of radical scavenging should be implemented during AEM fabrication.

## Conclusion

Through this study, we aimed to describe the initial processes of radical‐induced degradation of quaternary ammonium compounds (QAs), constituents of anion‐exchange membranes (AEMs). Pulse radiolysis experiments of benzylic‐type QAs indicate that the attack of HO⋅ at pH 7 or O⋅^−^ at pH 14 on QAs results in the formation of species that are oxidizing and reducing, with a lifetime in the order of milliseconds. We performed “redox titrations” to show that the yield of the reducing species is substantial. Steady‐state γ‐radiolysis experiments allowed us to establish that electron‐rich derivatives are more prone to radical‐induced degradation due to their higher susceptibility to hydrogen abstraction and may also readily undergo reductive deamination. This is important to consider during AEM development as current state‐of‐the‐art ionomers typically contain electron‐rich constituents. In comparison with aromatic proton‐exchange membranes, AEMs react with oxidizing radicals with lower rates, and therefore the use of radical scavengers may be a viable approach to prolong the lifetime of such ionomers. Our findings may be exploited for the design of AEMs that are stable against both highly alkaline conditions and radical attack.

## Experimental Section

### Chemicals and reactants

Halide salts of BTM, BMP, MBTM, or NBTM were synthesized according to the literature procedure,^[20]^ and then were converted to perchlorate salts by treating the aqueous solutions of the respective halide salts by HClO_4_ or AgCl_4_. Purity of the compounds was evaluated using an Agilent 1200 series HPLC system (Agilent, USA), gradient elution of AcN/H_2_O solvent system containing 0.025 % HCOOH acidic additive (10 μL injection, Waters μBondapak C18 Column, 125 Å, 10 μm, 3.9 mm×300 mm, Part No. WAT027324) and was found to be >99 %. The same system was used to evaluate the degree of degradation following the continuous irradiation experiments. Ultra‐pure water (18.2 MΩ cm, TOC<5 ppb) was provided by a Milli‐Q or Evoqua Ultra Clear UV Plus water purification system. Organic solvents were of HPLC grade. ABTS^2−^, TMPD, and MV^2+^ were used as received.

### Pulse radiolysis study

Experiments were carried out with a 2 MeV Febetron 705 accelerator (L‐3 Communications, San Leandro, CA). The equipment delivered <50 ns pulses of 2–100 Gy. Absolute doses were determined by KSCN dosimetry, based on *G*=0.635 μmol J^−1^ and *ϵ*_472_=7580 m ^−1^ cm^−1^, where 1 Gy=1 J kg^−1^ or in water around 1 J L^−1^. Samples were gas‐saturated in Schlenk‐tubes sealed with rubber septa, which were repeatedly evacuated to 10 mbar and refilled (a minimum of 3 repeats) with the desired gas. The solutions then were transferred to a gas‐tight syringe (10 mL, Hamilton, SampleLock, Bonaduz, Switzerland), which was connected to the 6 cm quartz irradiation‐cell (Hellma, Mülhausen, Germany) via a syringe pump. Neutral pH was established using 10 mm potassium phosphate buffer (KPi). Alkaline pH was established using 0.1 or 1 m KOH. Experiments were carried out at room temperature (25 °C). Uncertainties given represent standard deviation for a minimum of 3 experiments. Typically, a 10 % uncertainty is associated with the dose. The identity of the oxidizing radicals was evaluated using ABTS^2−^ [*E* ^0^(ABTS⋅^−^/ABTS^2−^)=0.68 V].[^23^, ^24^] The formation of ABTS⋅^−^ was monitored at one of its absorbance maxima, 650 nm, where only ABTS⋅^−^ absorbs with *ϵ*_650_=13000 m ^−1^ cm^−1^. Analogously, weakly oxidizing radicals were studied using TMPD [*E* ^0^(TMPD/TMPD⋅^+^)=0.276 V]. The formation of TMPD was followed at one of its absorbance maxima, 612 nm, where only TMPD⋅^+^ absorbs with *ϵ*_612_=12000 m ^−1^ cm^−1^.^[25]^ The radiation chemical yields of reducing radicals were determined using methyl viologen [*E* ^0^(MV^2+^/MV⋅^+^)=‐0.448 V].^[17]^ Formation of MV⋅^+^ was evaluated at 600 nm, where only MV⋅^+^ absorbs with *ϵ*_600_=11850 m ^−1^ cm^−1^.^[26]^

### Continuous irradiation experiments

For continuous radiolysis, samples were exposed to 110 Gy h^−1 60^Co γ‐radiation at the irradiation facility of PSI (Gammacell 220, Atomic Energy of Canada Limited). N_2_O‐, air‐, or argon‐saturated samples were prepared using a Schlenk‐line (N_2_O and air) or in a glovebox placed under argon.

### BDE calculations

Calculations were performed using the Gaussian 13 software suite.^[38]^ We performed the calculations at the ωB97XD/6‐31G(d) level of theory, which is known to produce reliable results with low computational requirements.^[39]^ Geometry optimizations were performed using the standard 6–31G(d) basis set. The calculated values are given in vacuum at room temperature. All optimizations were checked for convergence to an energy minimum, including probing for proper termination of Gaussian and ensuring that the resulting structure had no imaginary vibrational frequencies.

## Author Contributions

T. Nemeth synthesized, purified and characterized the model compounds, was responsible for the curation and analysis of data, and developed the methodology used in the degradation studies. T. Nauser conceptualized this study and, together with T. Nemeth, was responsible for the design of the experiments and the writing of the manuscript. L. Gubler was essential for the conceptualization of the project, securing funds, for project supervision and the writing of the manuscript.

## Conflict of interest

The authors declare no conflict of interest.

1

## Supporting information

As a service to our authors and readers, this journal provides supporting information supplied by the authors. Such materials are peer reviewed and may be re‐organized for online delivery, but are not copy‐edited or typeset. Technical support issues arising from supporting information (other than missing files) should be addressed to the authors.

Supporting Information

## Data Availability

The data that support the findings of this study are available from the corresponding author upon reasonable request.
