# Supplementary material for: On the Radical‐Induced Degradation of Quaternary Ammonium Cations for Anion‐Exchange Membrane Fuel Cells and Electrolyzers
Source: ChemSusChem. 2022 Oct 20;15(22):e202201571. doi: 10.1002/cssc.202201571 (PMC9828592; doi:10.1002/cssc.202201571)
Supplement: Supplementary file 1 — Supporting Information [file CSSC-15-0-s001.pdf]

ChemSusChem

Supporting Information

On the Radical-Induced Degradation of Quaternary Ammonium Cations for Anion-Exchange Membrane Fuel Cells and Electrolyzers

Tamas Nemeth,* Thomas Nauser, and Lorenz Gubler© 2022 The Authors. ChemSusChem published by Wiley-VCH GmbH. This is an open access article under the terms of the Creative Commons Attribution License, which permits use, distribution and reproduction in any medium, provided the original work is properly cited.

Table of contents:

1. Transient absorbance spectra of BMP, MBTM and NBTM
2. Kinetics experiments
3. Competing reactions in the pulse radiolysis experiments
4. Radiochemical yield of $ABTS^{\bullet -}$, $TMPD^{\bullet +}$ and $MV^{\bullet +}$

1. Transient absorbance spectra of BMP, MBTM and NBTM

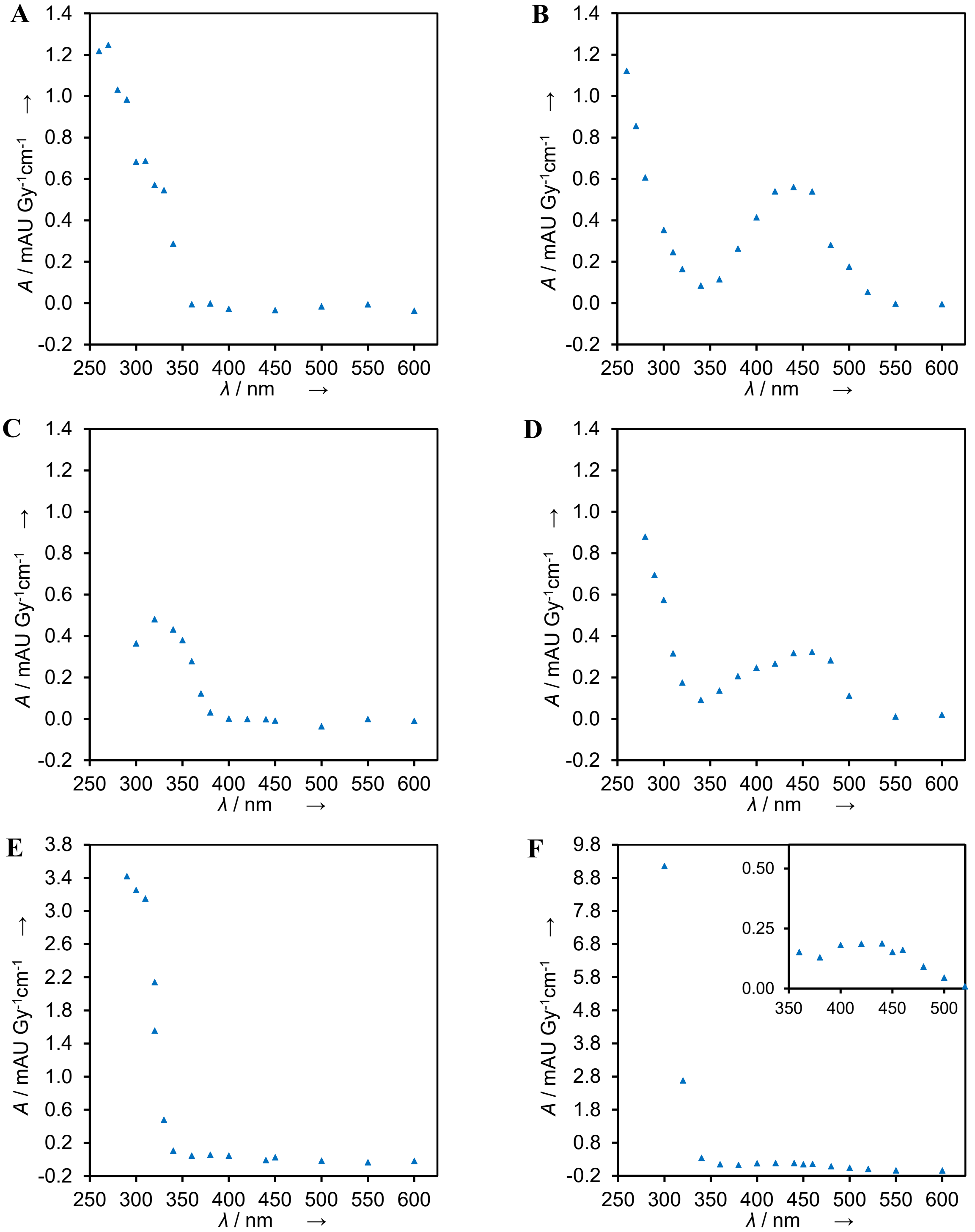

Figure S1. Transient absorption spectra (blue triangles) 100 μs after the pulse (dose 5–20 Gy), obtained from time-resolved absorbance readings, normalized to 1 Gy and to the optical path length, measured in N_2O-saturated aqueous solutions that contained 0.1 mM BMP and 10 mM KPi buffer (A) or 0.1 mM BMP and 100 mM KOH (B) or 0.1 mM MBTM and 10 mM KPi buffer (C) or 0.1 mM MBTM and 100 mM KOH (D) or 0.1 mM NBTM and 10 mM KPi buffer (E) or 0.1 mM NBTM and 100 mM KOH (F).

2. Kinetics experiments

Fig. S2 shows the slow decay of absorbance at 450 nm recorded for the pulse irradiated N_2O-saturated solutions of 1 mM BTM that contained 100 mM KOH.

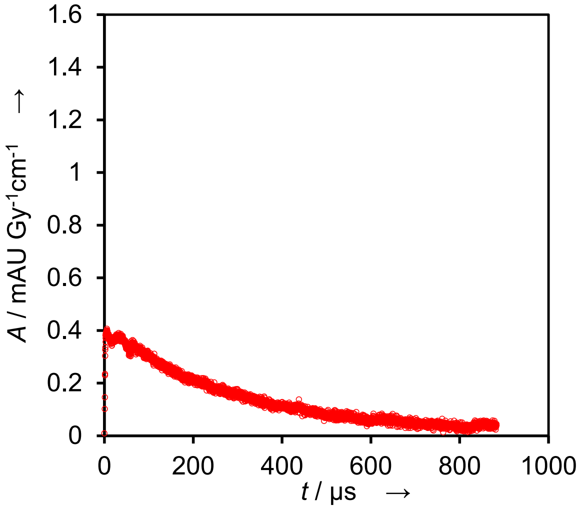

Figure S2. Change of optical density at 450 nm within the first 1000 µs after the pulse, observed in irradiated (dose 3-10 Gy) N_2O- saturated solutions of 1 mM BTM that contained 100 mM KOH (red circles). Normalized to the optical path length and to the maximum absorbance, recorded at room temperature.

Fig. S3 depicts the rapid build-up of absorbance at both neutral and alkaline conditions indicating that reactions involving both $O^{\bullet -}$ and $HO^{\bullet}$ were completed in ~3 µs.

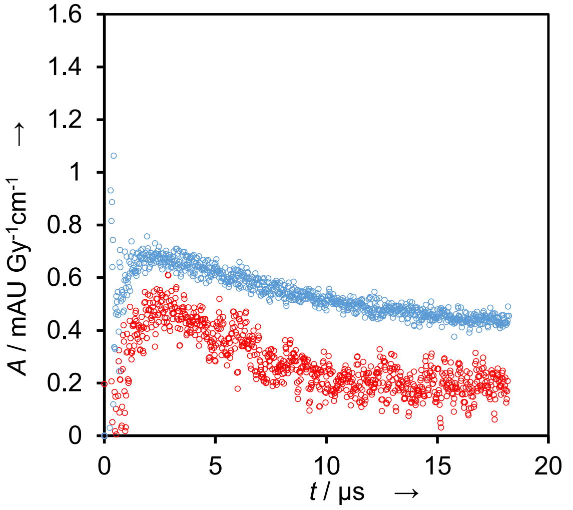

Figure S3. Change of optical density at 320 nm within the first 20 µs after the pulse, observed in irradiated (dose 3-10 Gy) N_2O- saturated solutions of 1 mM BTM that contained 10 mM KPi buffer (blue circles) or 100 mM KOH (red circles). Normalized to the optical path length and to the maximum absorbance, recorded at room temperature.

3. Competing reactions in the pulse radiolysis experiments

Neutral pH

Numbering of the reactions corresponds to the main text of the manuscript.

In case of argon-saturation, the yield of primary radicals produced by γ-radiolysis of water is $G(HO^•) = G(e_{aq}^-) = 0.28$ µmol J^{-1} and $G(H^•) = 0.06$ µmol J^{-1}, reaction (1).[1] The radiochemical yields will change if instead of argon N_2O or air is used.

In N_2O, hydrated electrons will form $HO^•$, reaction (2),[1] or react with the AEM model compounds through reductive dealkylation, reactions (10) and (11).

The solubility of N_2O is 24.8 mM and we irradiated 1 mM of model compounds in the degradation study, therefore, only 4.3 % of hydrated electrons is expected to react via reaction (10) or (11), while 95.7 % will react with N_2O. The yield of $HO^•$ is increased to $G(HO^•) = 0.28 + 0.27 = 0.55$ µmol J^{-1}, while $G(H^•)$ remains unchanged (0.06 µmol J^{-1}).

$N_2O + H_2O + e_{aq}^- \rightarrow N_2 + HO^• + OH^-$	(2)	$k=9.1\times10^9$ M^{-1}s^{-1}
$ArCH_2NR_3^+ + e_{aq}^- \rightarrow ArCH_2NR_2 + R^•$	(10)	$k=1.0\times10^{10}$ M^{-1}s^{-1}
$ArCH_2NR_3^+ + e_{aq}^- \rightarrow ArCH_2^• + NR_3$	(11)	$k=1.0\times10^{10}$ M^{-1}s^{-1}

In case of air-saturation, the biradical O_2 partially scavenges the available hydrated electrons, reaction (9). O_2 has a solubility of 0.26 mM, therefore $G(e_{aq}^-)$ is decreased by 32.9 % to 0.188 µmol J^{-1} and $G(O_2^{•-}) = 0.92$ µmol J^{-1}. $G(H^•)$ remains yet again unaltered (0.06 µmol J^{-1}).

$O_2 + e_{aq}^- \rightarrow O_2^{•-}$	(9)	$k=1.9\times10^{10}$ M^{-1}s^{-1}

Highly alkaline conditions

pH 14

At pH 14 we also have to consider reactions (3) and (4), all formed $HO^•$ and $H^•$ will deprotonate to $O^{•-}$ and e_{aq}^-, respectively. Therefore, under argon-saturation $G(O^{•-}) = 0.28$ µmol J^{-1} and $G(e_{aq}^-)$ changes to $0.28 + 0.06 = 0.34$ µmol J^{-1}.

Under N_2O-saturation, in the presence 1 mM AEM model compound, $G(O^{•-})$ is increased to $0.28 + (0.28 + 0.06) \times 0.96 = 0.61$ µmol J^{-1}.

In case of air-saturation the yield of hydrated electrons expected to react via reaction (10) or (11) is $G(e_{aq}^-) = 0.228$ µmol J^{-1}; therefore $G(O^{•-}) = 0.28$ µmol J^{-1} and $G(O_2^{•-}) = 0.112$ µmol J^{-1}.

$HO^{\bullet} + OH^{-} \rightarrow O^{\bullet -} + H_2O$ (3) $k=1.2\times10^{10}$ $M^{-1}s^{-1}$

$H^{\bullet} + OH^{-} \rightarrow e_{aq}^{-} + H_2O$ (4) $k=2.2\times10^{7}$ $M^{-1}s^{-1}$

pH 13

The pK_a of 11.8 for $HO^{\bullet}$ means that both its protonated (~10 %) and deprotonated form, $O^{\bullet -}$ (~90 %) will exist at pH 13. Therefore, under N_2O-saturation, in the presence 1 mM AEM model compound, the respective yields are $G(O^{\bullet -}) = 0.90\times(0.28+(0.06+0.28)\times0.96)) = 0.55$ μmol $J^{-1}$and $G(HO^{\bullet}) = 0.10\times(0.28+(0.06+0.28)\times0.96)) = 0.06$ μmol J^{-1}.

4. Radiochemical yield of $ABTS^{\bullet-}$, $TMPD^{\bullet+}$ and $MV^{\bullet+}$

In N_2O-saturated solutions of 1 mM AEM model compound, 0.1 mM ABTS and 100 mM KOH reaction (S1) and (S2) needs to be considered as well. ABTS reacts with $HO^\bullet$ with a rate constant of 1.2×10^{10} $M^{-1}s^{-1}$ and with $O^{\bullet-}$ with a rate constant of 2.54×10^9 $M^{-1}s^{-1}$.[2],[3]

$ABTS^{2-} + HO^\bullet \rightarrow ABTS^{\bullet-} + H_2O$ (S1) $k = 1.2 \times 10^{10}$ $M^{-1}s^{-1}$

$ABTS^{2-} + O^{\bullet-} \rightarrow$ Product (S2) $k = 2.54 \times 10^9$ $M^{-1}s^{-1}$

BTM and $HO^\bullet$ react with a rate constant of 5.0×10^9 $M^{-1}s^{-1}$, and with with $O^{\bullet-}$ with a rate constant of 5.9×10^8 $M^{-1}s^{-1}$.[4],[5] We assume that the rate constants for BMP, MBTM and NBTM have the same values. We show the estimation of yields below for BTM, we applied the same logic for the other model compounds as well.

In the presence of ABTS, radiochemical yields will change due to competing reactions (S1) and (S2). We expect the available $HO^\bullet$ that reacts with BTM to decrease to $G_{BTM + HO^\bullet}$ = 0.371 µmol J^{-1}, similarly available $O^{\bullet-}$ to react with BTM changes to $G_{BTM + O^\bullet-}$ = 0.048 µmol J^{-1}. We assume the products of these reactions to oxidize ABTS according to reaction (8), therefore $G_{ABTS^\bullet-,theoretical}$ = 0.371 + 0.048 = 0.419 µmol J^{-1}. In contrast to reaction (S2) that does not produce $ABTS^{\bullet-}$, reaction (S1) yields $ABTS^{\bullet-}$ with $G_{ABTS^\bullet-}$ = 0.011 µmol J^{-1}. The observed absorbance needs to be corrected accordingly, with the absorbance as measured in **Figure 4** and $\varepsilon_{650nm}(ABTS^{\bullet-})$ = 13000 $M^{-1}cm^{-1}$ we calculate $G_{ABTS^\bullet-}$ = 0.105 µmol J^{-1}. The yield of reaction (6) then is (0.105 – 0.011) / 0.419 = 22.3%. We consider the calculated yield as lower limit.

Analogously, in N_2O-saturated solutions of 1 mM AEM model compound, 0.1 mM TMPD and 100 mM KOH we have to consider reaction (S3) and (S4). TMPD reacts with $HO^\bullet$ with a rate constant of 3×10^8 $M^{-1}s^{-1}$ and with $O^{\bullet-}$ we assume the same rate constant of 3×10^8 $M^{-1}s^{-1}$.[6],[7] Applying the same methodology as above we estimate $G_{TMPD^\bullet+,theoretical}$ = 0.565 µmol J^{-1} for reaction (7). With the absorbance as measured in **Figure 5** and $\varepsilon_{612nm}(TMPD^{\bullet+})$ = 12000 $M^{-1}cm^{-1}$ we calculate $G_{TMPD^\bullet+}$ = 0.152 µmol J^{-1}.[7] The yield of reaction (7) then is 0.152 / 0.565 = 26.9%.

$TMPD + HO^\bullet \rightarrow TMPD^{\bullet+} + H_2O$ (S3) $k = 3 \times 10^8$ $M^{-1}s^{-1}$

$TMPD + O^{\bullet-} \rightarrow$ Product (S4) $k = 3 \times 10^8$ $M^{-1}s^{-1}$

When N_2O-saturated solutions of 1 mM AEM model compound, 0.1 mM MV^{2+} and 100 mM KOH are pulse irradiated we have to consider reaction (S5), (S6) and (S7). MV^{2+} reacts with $HO^\bullet$ with a rate constant of 2.5×10^8 $M^{-1}s^{-1}$ and with $O^{\bullet-}$ with a rate constant of 1.5×10^9 $M^{-1}s^{-1}$.[1] MV^{2+} partially scavenges the available hydrated electrons with a rate constant of 5.5×10^{10} $M^{-1}s^{-1}$.[8]

$MV^{2+} + HO^\bullet \rightarrow$ Product (S5) $k = 2.5 \times 10^8$ $M^{-1}s^{-1}$

$MV^{2+} + O^{\bullet-} \rightarrow$ Product (S6) $k = 1.5 \times 10^9$ $M^{-1}s^{-1}$

$MV^{2+} + e_{aq}^- \rightarrow$ Product (S7) $k = 5.5 \times 10^{10}$ $M^{-1}s^{-1}$

We thus estimate $G_{MV^{\bullet+},theoretical}$ = 0.477 µmol J^{-1} stemming from reaction (8). With the absorbance as measured in **Figure 6** and $\varepsilon_{600nm}(MV^{\bullet+})$ = 11850 $M^{-1}cm^{-1}$ we calculate $G_{MV^{\bullet+}}$ = 0.231 µmol J^{-1}.[7] The yield of reaction (8) then is 0.231 / 0.477 = 48.4%.

REFERENCES

[1] G. V. Buxton, C. L. Greenstock, W. P. Helman and A. B. Ross, *J. Phys. Chem. Ref. Data*, **1988**, *17*, 513.

[2] B. S. Wolfenden and R. L. Willson, *J Chem Soc Perkin Trans II*, **1982**, 805–812.

[3] L. Szabó, V. Mile, T. Tóth, Gy. T. Balogh, T. Földes, E.Takács and L. Wojnárovits, *Free Radic. Res.*, **2017**, *51(2)*, 124–140.

[4] A. Kabi and P. G. Clay, *Radiat. Res.*, **1968**, *34*, 680–688.

[5] K. Bobrowski, *J. Phys. Chem.*, **1981**, *85(4)*, 382–388.

[6] M.N. Schuchmann,M.L. Scholes,H. Zegota and C. Von Sonntag, *Int. J. Radiat. Biol.*, **1995**, *68*, 121–131.

[7] S. Steenken, A. J. S. C. Vieira, *Angew. Chem. Int. Ed.*, **2001**, *40(3)*, 571–573.

[8] A. J. Elliot, D. R. McCracken, G. V. Buxton and N. D. Wood, *J. Chem. Soc. Faraday. Trans.*, **1990**, *86*, 1539–1547.
